# Role of Plant Growth-Promoting Bacteria in Reshaping Rhizosphere Bacterial and Fungal Microbiomes Under Multi-Metal–Microplastic Composite Pollution in Spinach

**DOI:** 10.3390/microorganisms14050972

**Published:** 2026-04-26

**Authors:** Xiao-Lu Luo, Jing-Yi Wang, Yan-Qin Tang, Ze-Hua Hu, Han Liu, Bai-Lian Larry Li, Yu-Ying Li, Xue-Min Ren, Hui Han, Yan Chen, Zhao-Jin Chen

**Affiliations:** 1 School of Water Resource and Modern Agriculture, Nanyang Normal University, Nanyang 473061, China; 2 College of Resources and Environment, Yangtze University, Jingzhou 434020, China; 3 Overseas Expertise Introduction Center for Discipline Innovation of Watershed Ecological Security in the Water Source Area of the Mid-line Project of South-to-North Water Diversion, Nanyang 473061, China

**Keywords:** Cd, Pb, PLA microplastic, plant growth-promoting bacteria, rhizosphere microbiome

## Abstract

Microplastics (MPs) often co-occur with heavy metals (HMs), posing combined stress that inhibits plant growth. While plant growth-promoting bacteria (PGPB) are known to alleviate heavy metal toxicity, their role under MP–HM co-contamination and the differential responses of rhizosphere microbial communities remain unclear. This study evaluated the effects of cadmium (Cd) and lead (Pb), polylactic acid (PLA) MPs, and their combined contamination on spinach growth using pot experiments, and assessed the mitigation potential of two PGPB strains. PGPB inoculation significantly increased plant height and dry weight. High-throughput sequencing revealed that pollution treatments and PGPB altered rhizosphere bacterial and fungal community composition and diversity. Microbial shifts were closely associated with soil chemical properties and plant growth. Notably, bacteria and fungi exhibited distinct response patterns to combined stress and remediation. Functional prediction (PICRUSt2) indicated that microbial communities enhanced metabolic processes and nutrient (N and P) cycling to cope with stress. PGPB inoculation reduced heavy metal toxicity, improved soil nutrient status (P and K), increased microbial diversity, and regulated microbial functions, thereby supporting soil ecological stability. These findings provide insights into rhizosphere microbial mechanisms and support the application of PGPB for remediation of MP–HM co-contaminated soils.

## 1. Introduction

Plastic products are widely used in consumer goods and industrial applications, leading to substantial amounts of plastic entering the environment after use due to their persistence [1]. Microplastics (MPs), defined as particles smaller than 5 mm, have been recognized as emerging pollutants originating from consumer products, industrial raw materials, and the fragmentation of larger plastic debris [2]. Due to their widespread occurrence and associated environmental risks, MPs have attracted increasing attention. A recent nationwide survey conducted in 2025 reported that MPs were detected at 79.7% of 1331 sampling sites across China [3]. MPs in soil originate from multiple sources, including organic fertilizer application, plastic mulching, and atmospheric deposition [4]. MPs have been shown to disrupt soil structure, alter physicochemical properties, and inhibit plant growth [5,6]. In addition, MPs can influence soil microbial communities, posing potential risks to ecosystem stability. Therefore, further investigation into the impacts of MPs on soil–plant–microbe interactions is warranted.

With the rapid development of industrialization and urbanization, heavy metal pollution has become a major threat to ecological systems and human health [7]. Heavy metals (HMs) exhibit a high degree of persistence in the environment, accumulating through the food chain and posing a significant threat to human health [8]. Among them, cadmium (Cd) and lead (Pb) are particularly prevalent and highly toxic environmental pollutants [9,10]. According to the National Soil Pollution Status Survey Bulletin, Cd and Pb exceeded standard limits at 7% and 1.5% of sampling sites, respectively [11]. For example, in certain regions of Guangdong Province, informal recycling of waste batteries, including incineration for metal recovery, has released large quantities of Cd- and Pb-containing emissions and wastewater, resulting in severe environmental contamination and health risks [12]. This resulted in severe local environmental pollution and posed serious health risks to the local population.

In environmental systems, MPs frequently co-occur with HMs, forming complex composite pollutants [13]. Due to their large specific surface area and diverse functional groups, MPs exhibit strong adsorption and transport capacities [14]. These properties enable MPs to adsorb HMs and act as carriers, facilitating their migration and bioavailability [15]. Previous studies have demonstrated that MPs can adsorb metals such as Cu, Cd, and Pb, thereby enhancing their ecological risks [16,17]. Moreover, degradation of biodegradable MPs such as Polybutylene Adipate Terephthalate (PBAT) can decrease soil pH, thereby increasing Pb solubility and exacerbating pollution stress on plants and soil ecosystems [18,19]. Given the increasing prevalence of such composite pollution, the remediation of MP–HM complexes remains a significant challenge, highlighting the need for innovative synergistic remediation strategies.

Plant-growth-promoting bacteria (PGPB) are defined as beneficial microorganisms that colonize plant roots or internal tissues [20]. PGPB can promote plant growth and enhance stress tolerance through various direct and indirect mechanisms, making them promising agents for the remediation of complex contaminated soils [21,22,23]. Previous studies have demonstrated that PGPB can enhance plant tolerance to HMs and mitigate MP–HM co-contamination through multiple mechanisms, including improving soil nutrient availability, regulating microbial communities, altering plant gene expression, and enhancing antioxidant enzyme activity [24,25,26,27,28,29,30,31,32]. For instance, Liu et al. [33] reported that *Bacillus* sp. SL-413 and *Enterobacter* sp. VY-1 could regulate rhizosphere microbial community composition and interactions under MP–heavy metal stress, thereby alleviating plant stress. This process has been shown to alleviate stress experienced by plants in these environments. This demonstrates the practical potential of mitigating MP–heavy metal composite pollution stress by regulating the rhizosphere microbiome. It is noteworthy that the majority of these studies concentrate on PGPB remediation efforts in single heavy metal contamination and MP formation scenarios. However, most existing studies have focused on single heavy metal contamination or simplified MP systems, whereas multi-metal co-contamination commonly occurs in real soils [34]. Therefore, further research is needed to evaluate the effectiveness of PGPB in mitigating MPs combined with multi-heavy metal pollution. In addition, fungi, as key components of the rhizosphere microbiome, also play important roles in the response to and remediation of MP–HM stress [35]. Nevertheless, most studies have primarily focused on bacterial communities, while the coordinated responses of bacterial and fungal microbiomes remain poorly understood.

To address these knowledge gaps, this study employed a pot experiment to investigate: (i) the effects of single and combined Cd and Pb in combination with polylactic acid (PLA) MPs on spinach growth; (ii) the potential of PGPB to alleviate Cd + Pb + PLA-induced stress; and (iii) the mechanisms by which PGPB regulate rhizosphere bacterial and fungal communities under composite pollution. By integrating plant physiological traits, soil physicochemical properties, and high-throughput sequencing, this study provides new insights into how PGPB alleviate multi-heavy metal–MP stress, enhance plant growth, and modulate rhizosphere microbial communities.

## 2. Materials and Methods

### 2.1. Experimental Materials

The spinach seeds utilised in this experiment were procured from Shouguang Xinxinran Horticulture Co., Ltd (Shouguang, China). Seeds exhibiting uniform size, plump grains, and undamaged exteriors were selected for subsequent research. The MPs utilised in the experiment were PLA particles with a diameter of 100 μm, procured from the Dongguan Plastic Trading Department (Dongguan, China). The test bacterial strains were SQ6 and VY7, which had previously been subjected to laboratory screening in order to ascertain their capacity to promote growth, as reported by Zhang et al. [26] and Liu et al. [33]. The application of sequence alignment and phylogenetic tree analysis facilitated the identification of SQ6 as belonging to the genus *Bacillus* sp., while VY7 was classified as *Enterobacter* sp. Both strains possess the ability to produce siderophores, synthesize indole-3-acetic acid (IAA), and solubilize phosphorus, while exhibiting tolerance to Cd. The experimental soil was collected from the vicinity of the Rose Garden at Nanyang Normal University in Nanyang, China.

Soil samples were collected from the 0–20 cm cultivation layer, any impurities removed, and the samples air-dried under natural conditions. The soil was then ground into fine particles, passed through a 20-mesh sieve, and stored for later use.

### 2.2. Experimental Methods

The screened soil for pot treatment was mixed separately with CdSO_4_·8H_2_O and Pb(NO_3_)_2_, achieving Cd concentration of 10 mg·kg^−1^ and Pb concentration of 200 mg·kg^−1^. After thorough mixing to ensure uniform distribution, the soil was air-dried for one month to immobilize heavy metals. The experimental design incorporated treatments with two heavy metals, Cd and Pb, with MP PLA added at a concentration of 0.5%. Furthermore, a composite pollution treatment group combining Cd, Pb, and PLA was established, with the plant growth-promoting bacterial strains *Bacillus* sp. SQ6 and *Enterobacter* sp. VY7 incorporated into the composite pollution group. Concomitantly, a control group lacking intervention was established. The specific grouping details are shown in Table 1. Each group comprised three replicates, with three plants retained per pot. Each pot contained 0.75 kg of pretreated soil, into which spinach seeds were sown. During the cultivation process, the soil moisture levels were maintained through regular watering, and the pots were arranged in a random configuration with periodic rotation. Upon attaining a specified growth stage, the selection of plants was based on their resemblance in terms of vigour and vitality. This ensured that each pot contained three plants.

The strains that demonstrated significant growth-promoting properties, as identified through screening, were inoculated into liquid Lysogeny Broth medium (LB) and subsequently cultured under shaking conditions at 28 °C for a period of 12–24 h. A portion of the culture was transferred to a 50 mL centrifuge tube and subjected to centrifugation at 4800 rpm for 20 min, with the objective of collecting the bacterial cells. The cells were then subjected to several washes with sterile deionised water, and were subsequently resuspended at a concentration of 1 × 10^8^ CFU·mL^−1^ for subsequent utilisation. At 30, 45 days after seedling emergence, 5 mL of the bacterial suspension was applied to the root zone of each plant. A blank control was subjected to the same volume of sterile deionised water.

### 2.3. Pot Experiment and Sample Collection

Plant and rhizosphere soil samples were collected at 75 days after sowing. The soil adhering to the plant surfaces was meticulously rinsed with deionized water, thoroughly dried, and subsequently separated into aboveground and belowground components for the purpose of length measurement. The soil surrounding the roots was collected by means of shaking, followed by rinsing the plant roots with water. Plants were placed in envelopes and dried in an oven set at 72 °C for 24 h before determining dry weight.

The aboveground and belowground parts were ground into powder. The Cd and Pb concentration was determined by weighing 0.05 g of air-dried plant tissues, which were placed in Teflon tubes, followed by the addition of 5 mL of concentrated acid (HCl/HNO_3_ = 1:3, *v*/*v*). The tubes were then subjected to microwave digestion under controlled temperature and pressure. After digestion, the samples were filtered through quantitative filter paper and diluted with ultrapure water to a final volume of 50 mL. The Cd and Pb contents in plant tissues were determined using ICP-OES (Optima 2100 DV, PerkinElmer, MA, USA). The soil samples were air-dried naturally and then sieved through a 1 mm mesh. Each soil sample was weighed to an accuracy of 0.01 g, with at least 5 g used for analysis, and placed into a 25 mL centrifuge tube. Subsequently, 20 mL of DTPA–CaCl_2_–TEA extraction solution (c(DTPA) = 0.005 mol·L^−1^, c(CaCl_2_) = 0.01 mol·L^−1^, c(TEA) = 0.1 mol·L^−1^) was added, and the tube was sealed. The mixture was shaken at 25 °C for 2 h at 160–200 rpm. The extract was then centrifuged for 10 min, and the DTPA-extractable Cd and Pb contents in the supernatant were determined using ICP-OES.

Total phosphorus (TP) was determined using the NaOH fusion-molybdenum antimony colorimetric method, and available phosphorus (AP) was measured by extraction with 0.05 mol·L^−1^ HCl–0.025 mol·L^−1^ H_2_SO_4_ followed by molybdenum–antimony colorimetry. Total potassium (TK) was analyzed using the NaOH fusion–flame photometric method, whereas available potassium (AK) was determined by NH_4_OAc extraction followed by flame photometry [36].

### 2.4. High-Throughput Sequencing and Bioinformatics Analysis

Soil DNA extraction was performed using a slightly modified version of the standard protocol for the FastDNA Spin Kit for Soil (MP Biomedicals, OH, USA). High-throughput sequencing of the soil samples was conducted by Shanghai Majorbio Bio-Pharm Technology Co., Ltd. (Shanghai, China). Soil genomic DNA was extracted and checked for quality using 1% agarose gel electrophoresis. The 16S rRNA V3–V4 region of bacteria was amplified using primers 341F (5′-CCTACGGNGGCWGCAG-3′) and 805R (5′-GACTACHVGGGTATCTAATCC-3′). Fungal ITS1 regions were amplified using primers ITS1F (5′-CTTGGTCATTTAGAGGAAGTAA-3′) and ITS2R (5′-GCTGCGTTCTTCATCGATGC-3′) [37].

PCR was conducted in 20 μL reactions using a TransStart FastPfu DNA polymerase system under standard cycling parameters. Triplicate PCR reactions were performed per sample. Both bacterial and fungal amplifications involved two rounds of PCR. In the first round, we used 16S V3–V4 primers for bacteria and ITS1-ITS2 primers for fungi, with a 30 µL reaction mix (15 µL 2 × Hieff^®^ Robust PCR Master Mix, 1 µL of each primer, 10–20 ng of PCR products, and 9–12 µL of H_2_O). The PCR conditions were an initial denaturation at 94 °C for 3 min, followed by 5 cycles at 94 °C for 20 s, annealing at 45 °C for 20 s, and extension at 65 °C for 30 s; then 20 cycles at 94 °C for 20 s, 55 °C for 20 s, and 72 °C for 30 s, with a final extension at 72 °C for 5 min and storage at 10 °C. In the second round, Illumina bridge PCR-compatible primers were used with a similar reaction mix and conditions: pre-denaturation at 95 °C for 3 min, followed by 5 cycles of denaturation at 94 °C for 20 s, annealing at 55 °C for 20 s, and extension at 72 °C for 30 s, concluding with a final extension at 72 °C for 5 min and storage at 10 °C. PCR products were verified by 2% agarose gel electrophoresis, purified using the AxyPrep DNA Gel Recovery Kit (Axygen Biosciences, Union City, CA, USA), and quantified with a QuantiFluor™-ST fluorometer (Promega, Madison, WI, USA). Equimolar amplicons were pooled and subjected to paired-end sequencing on the Illumina MiSeq platform (MiSeq, Illumina, San Diego, CA, USA).

Raw sequencing data were spliced, quality-controlled using FASTP and FLASH, and filtered using QIIME2. Denoising, error estimation, chimera removal and merging were performed using DADA2. Amplicon sequence variants (ASVs) constructed by DADA2 were then clustered into operational taxonomic units at a 97% similarity cut off by UPARSE [38]. The SILVA database (version 138.1) was used for the identification of bacterial community composition, while the UNITE database (version 8.0) was used for the identification of fungal community composition. Microbial α-diversity and richness were calculated using Mothur 1.43.0. The analysis entailed the execution of Principal Component Analysis (PCA), redundancy analysis (RDA) and Mantel tests, for which the R software (version 4.2.0) was employed. Bacterial functional profiles were predicted using PICRUSt2 (version 2.2.0) based on the KEGG database. Fungal functions were annotated using MetaCyc pathway analysis and trophic mode classification via FUNGuild.

### 2.5. Statistical Analysis

All measurements were based on three biological replicates. Statistical analyses were performed using SPSS 23.0. One-way ANOVA, independent-sample *t*-tests, and two-way ANOVA with Duncan’s multiple range test were applied to assess differences among treatments. Significance was set at *p* < 0.05.

## 3. Results

### 3.1. Effects of Different Treatments on Spinach Growth

**Figure 1:**
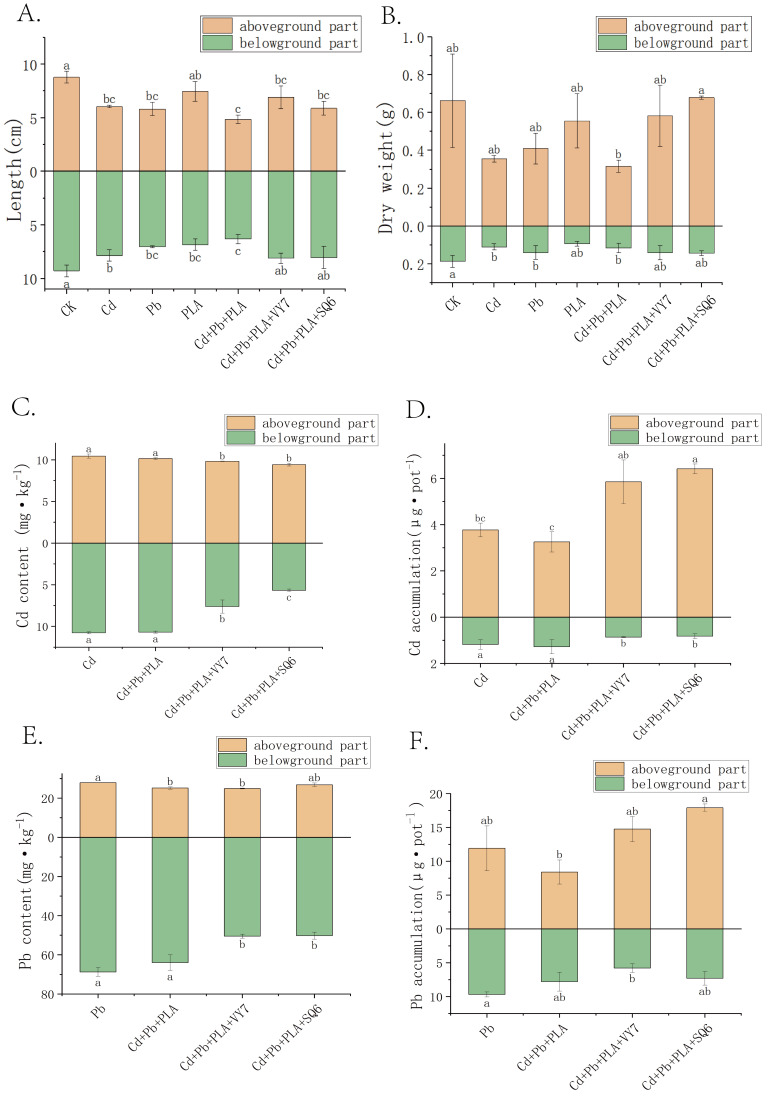
Length (**A**) and dry weight (**B**) of spinach under different treatment conditions. Cd content (**C**), Cd accumulation (**D**), Pb content (**E**), and Pb accumulation (**F**) in spinach under different treatment groups (mean ± SD). Different letters above or below the bars indicate significant differences (*p* < 0.05).

As shown in Figure 1A, compared to the CK group, shoot and root lengths of spinach in all pollution treatment groups decreased. Shoot length decreased by 15–45%, and root length decreased by 15–32%. The Cd+Pb+PLA co-contamination treatment group showed the most significant reduction in plant length. Compared to the Cd+Pb+PLA group, inoculation with SQ6 and VY7 increased shoot length by 21% and 42%, respectively, and root length by 28% and 29%, respectively.

As shown in Figure 1B, the effects of various pollution treatments and PGPB inoculation on plant dry weight were similar to those on length. Cd+Pb+PLA co-contamination, compared to single metal (Cd or Pb) or PLA pollution groups, caused a decrease in shoot and root dry weight. Compared to CK, the Cd+Pb+PLA group showed a 52% and 38% reduction in shoot and root dry weight, respectively. The Cd+Pb+PLA+SQ6 and Cd+Pb+PLA+VY7 treatment groups alleviated the co-contamination stress on plant growth, significantly increasing shoot dry weight by 115% and 84%, respectively, and root dry weight by 17–20%.

### 3.2. Effects of Different Treatments on Cd and Pb Content and Accumulation in Spinach

Compared to the single Cd treatment group, the Cd+Pb+PLA group showed reduced Cd content, with shoot and root Cd content decreasing by 3% and 1%, respectively (Figure 1C). PGPB inoculation significantly reduced plant Cd content. Inoculation with strain SQ6 decreased shoot and root Cd content by 6% and 29%, respectively, while strain VY7 decreased them by 10% and 47%, respectively. The effects of Cd+Pb+PLA co-contamination and PGPB inoculation treatments on Pb content were similar to those on Cd (Figure 1E). The Cd+Pb+PLA group reduced plant Pb content compared to the single Pb pollution group. PGPB strains SQ6 and VY7 also lowered plant Pb content. Analysis of plant Cd accumulation showed that PGPB inoculation significantly increased shoot Cd accumulation (80~97%) but significantly reduced root Pb accumulation (6~26%), demonstrating differential effects on different heavy metals (Figure 1D,F). The increased Cd and Pb accumulation in plants under PGPB inoculation may be attributed to a biomass dilution effect, where enhanced plant growth results in greater total metal uptake but reduced concentration. However, this raises important concerns regarding food safety, as increased total accumulation in edible tissues may pose potential risks.

### 3.3. Effects of Different Treatments on Rhizosphere Soil Physicochemical Properties and Available Metal Content

As shown in Table 2, compared to the CK group, single Cd and Pb pollution treatment groups showed decreasing trends in soil available phosphorus (AP), available potassium (AK), and total phosphorus (TP) content, with reductions of 5~12%, 18~34%, and 16~37%, respectively. PLA treatment increased AP and AK content but decreased TP and total potassium (TK) content. The Cd+Pb+PLA co-contamination group had significantly lower AK content compared to single pollution treatments, while TP and TK content were higher. Inoculation with strain VY7 significantly increased AP content by 28%; strains VY7 and SQ6 significantly increased AK content by 20% and 16%, respectively. Cd+Pb+PLA co-contamination increased soil DTPA-extractable Cd and Pb content by 19% and 12%, respectively. Strains VY7 and SQ6 increased soil DTPA-Cd content by 13.7% and 13.1%, respectively, but decreased DTPA-Pb content by 2.8% and 0.9%, respectively.

### 3.4. Bacterial and Fungal Community Diversity Indices

**Figure 2:**
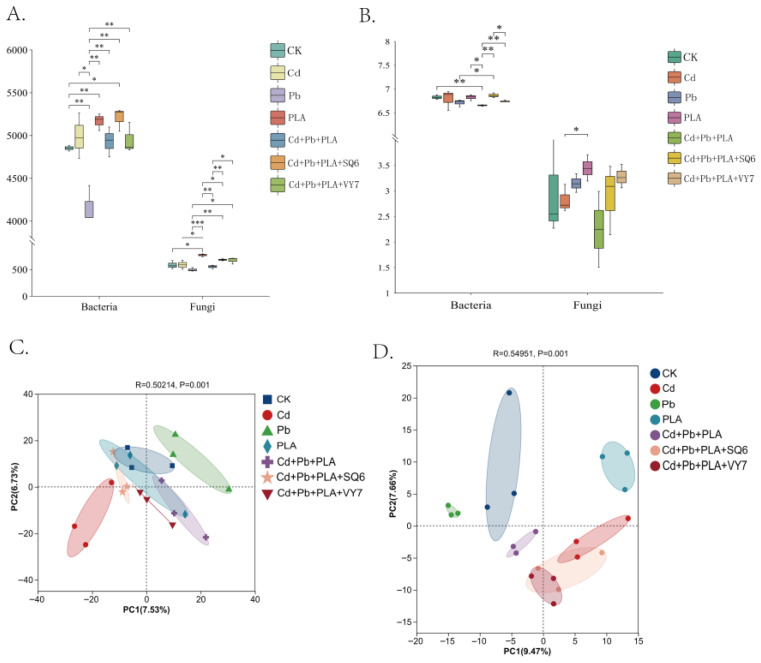
Diversity indices of microbial communities. (**A**) Comparison of ACE of bacterial and fungal community; (**B**) Comparison of Shannon of bacterial and fungal community. PCA chart of bacterial (**C**) and fungal (**D**) in the rhizosphere soil of spinach.*, *p* < 0.05; **, *p* < 0.01; ***, *p* < 0.001.

High-throughput sequencing results indicated a high coverage of microbial diversity (coverage > 99%). The responses of bacterial and fungal communities to pollution were similar. Compared to CK, the single Cd treatment group showed increased ACE indices, while the single Pb group showed decreased ACE indices (Figure 2A). The single PLA treatment group had significantly higher ACE indices. As shown in Figure 2A, PGPB inoculation increased community diversity. The addition of both PGPB strains SQ6 and VY7 increased the ACE indices of both bacterial and fungal communities. The Shannon index of bacterial communities was higher than that of fungal communities, ranging from 6.63 to 6.94 for bacteria and 1.51 to 4.07 for fungi (Figure 2B). The Cd+Pb+PLA co-contamination group reduced the Shannon index of microbial communities, while PGPB inoculation increased the Shannon indices of both bacteria and fungi. The ACE and Shannon indices indicated that the diversity and richness of bacterial communities in spinach rhizosphere soil were higher than those of fungal communities.

### 3.5. Principal Component Analysis (PCA) of Bacterial and Fungal Communities

Figure 2 illustrates the distribution patterns of bacterial and fungal communities across the seven treatment groups, as revealed by PCA, and demonstrates that there are distinct differences in soil microbial community distribution among the different treatments. As illustrated in Figure 2C, within the bacterial community, the bacterial communities of the Cd and Pb groups exhibit clear separation from those of the remaining treatment groups. As demonstrated in Figure 2D, the distribution of fungal communities across the seven treatment groups is characterised by significant dispersion. Among these, the Cd+Pb+PLA group exhibits a more concentrated distribution compared to the Cd+Pb+PLA+SQ6 and Cd+Pb+PLA+VY7 groups, indicating higher species similarity among these three fungal communities. In comparison with bacterial communities, fungal communities exhibited greater dispersion, indicating that the differences in composition among the seven groups were more pronounced than the differences in bacterial composition.

### 3.6. Composition and Differential Analysis of Bacterial and Fungal Communities

**Figure 3:**
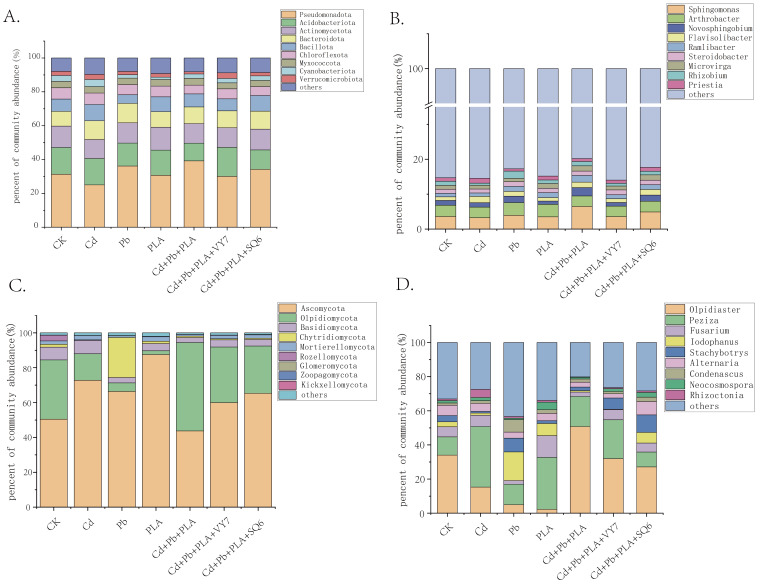
Relative abundance of microbial communities in different treatment groups at the phylum and genus levels. (**A**) bacterial phylum level; (**B**) bacterial genus level; (**C**) fungal phylum level; (**D**) fungal genus level.

High-throughput sequencing results showed that spinach rhizosphere bacteria comprised 46 phyla and 1186 genera. As shown in Figure 3A, at the phylum level, dominant phyla were *Pseudomonadota* (25.0–39.19%), *Acidobacteriota* (10.47–15.80%), *Actinomycetota* (11.24–12.64%), *Bacteroidota* (9.32–11.12%), and *Bacillota* (5.09–9.64%). At the genus level, the dominant bacterial genera were *Sphingomonas* (3.23–6.47%), *Arthrobacter* (3.00–3.55%), *Novosphingobium* (0.96–2.42%), *Flavisolibacter* (1.00–1.80%), and *Ramlibacter* (0.98–1.93%) (Figure 3B). Spinach rhizosphere fungi were classified into 16 phyla and 544 genera. Dominant fungal phyla were *Ascomycota* (43.69–87.48%), *Olpidiomycota* (2.17–50.75%), *Basidiomycota* (2.90–7.69%), *Chytridiomycota* (0.29–22.97%), and *Mortierellomycota* (0.91–2.58%) (Figure 3C). At the genus level, dominant fungal genera were *Olpidiaster* (2.17–50.75%), *Peziza* (8.74–35.43%), *Fusarium* (2.18–12.79%), and *Iodophanus* (0.22–16.70%) (Figure 3D).

Significant differential analysis of bacterial and fungal community taxa at the phylum and genus levels among the seven groups showed that among the top ten abundant bacterial phyla, four dominant phyla—*Bacillota*, *Fibrobacterota*, *Armatimonadota*, and *Ignavibacteriota*—exhibited significant differences (*p* < 0.05). Ten genera, including *Sphingomonas*, *Novosphingobium*, *Flavisolibacter*, and *Rhizobium*, also showed significant differences (*p* < 0.05). For fungal communities, all top ten abundant dominant genera showed significant differences, with *Iodophanus* and *Lobulomycetales_gen_Incertae_sedis* showing extremely significant differences (Figure 3).

### 3.7. Redundancy Analysis (RDA) of Rhizosphere Soil Bacterial and Fungal Communities

**Figure 4:**
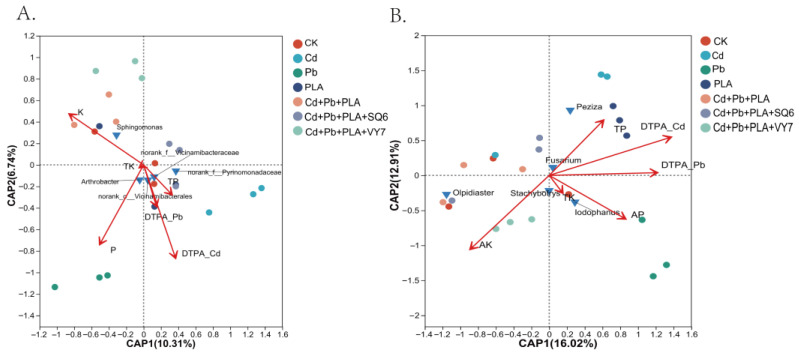
Redundancy analysis (RDA) of bacterial (**A**) and fungal (**B**) communities in relation to soil chemical characteristics. Red arrows indicateenvironmental variables, whereas blue triangles indicate dominant phyla.

Redundancy Analysis (RDA) was used to evaluate factors influencing bacterial and fungal community structures. As shown in Figure 4A, the first two axes explained 10.31% (CAP1) and 6.74% (CAP2) of the variation in bacterial communities. Bacterial community structure was significantly correlated with AP (R^2^ = 0.56034, *p* = 0.002), AK (R^2^ = 0.56661, *p* = 0.003), and DTPA-Cd (R^2^ = 0.55556, *p* = 0.002).

Figure 4B shows that the first two axes explained 16.02% (CAP1) and 12.91% (CAP2) of the variation in fungal communities. Fungal community structure was significantly correlated with AK (R^2^ = 0.50367, *p* = 0.04) and DTPA-Cd (R^2^ = 0.58314, *p* = 0.02). Comparing correlations between environmental factors and bacterial vs. fungal communities revealed stronger correlations between environmental factors and dominant bacterial genera, indicating that bacterial communities are more sensitive and directly responsive to environmental changes.

### 3.8. Functional Prediction for Bacteria and Fungi

**Figure 5:**
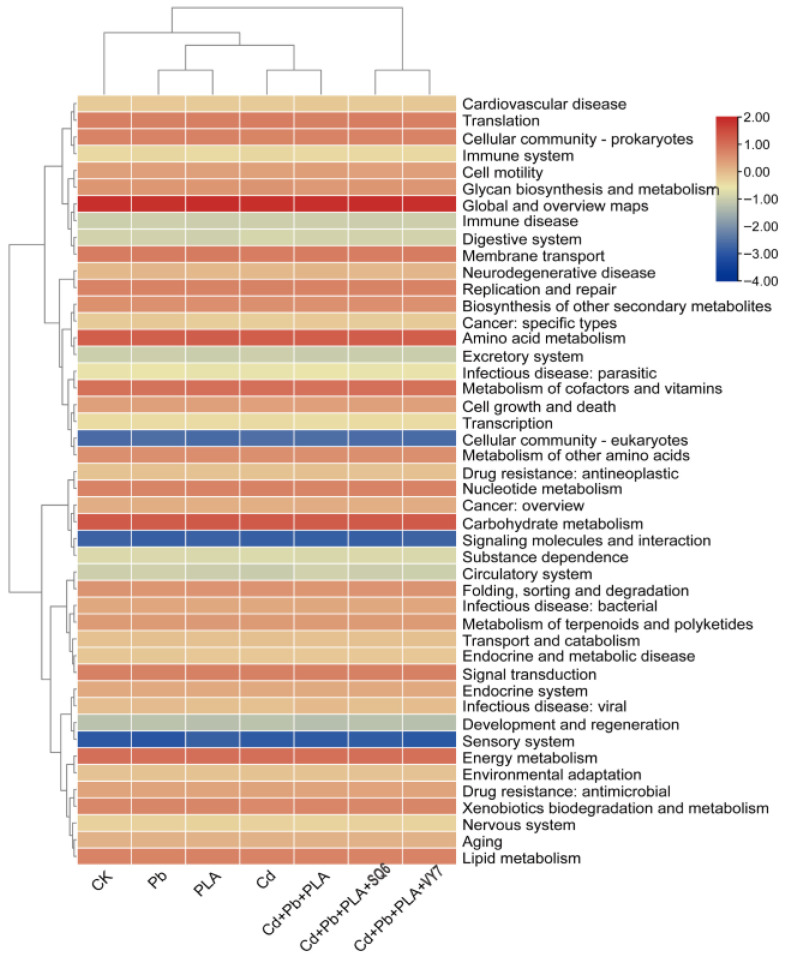
Heatmap of bacterial functions in different treatment groups. The *x*-axis represents treatment groups, and the *y*-axis represents functional genes.

To further investigate bacterial functional potential, PICRUSt2 was used to predict functional differences based on COG categories among treatment groups under different MP–HM pollution conditions. The predicted functions were classified into five major categories: Cellular Processes, Environmental Information Processing, Human Diseases, Metabolism, and Organismal Systems. As shown in Figure 5, the seven groups clustered into two main clusters: the PGPB-inoculated groups (Cd+Pb+PLA+SQ6 and Cd+Pb+PLA+VY7) formed one cluster, while the remaining five groups formed another. Among the non-PGPB groups, CK formed a separate sub-cluster, whereas the Cd group clustered with the Cd+Pb+PLA group. Compared with CK, the Cd treatment group showed an overall increase in the predicted relative abundance of functional genes. Compared with the Cd+Pb+PLA group, PGPB addition was associated with a decrease in the predicted abundance of genes related to glycan biosynthesis and metabolism and the digestive system, whereas other functional categories showed an increasing trend in their predicted gene abundance.

**Figure 6:**
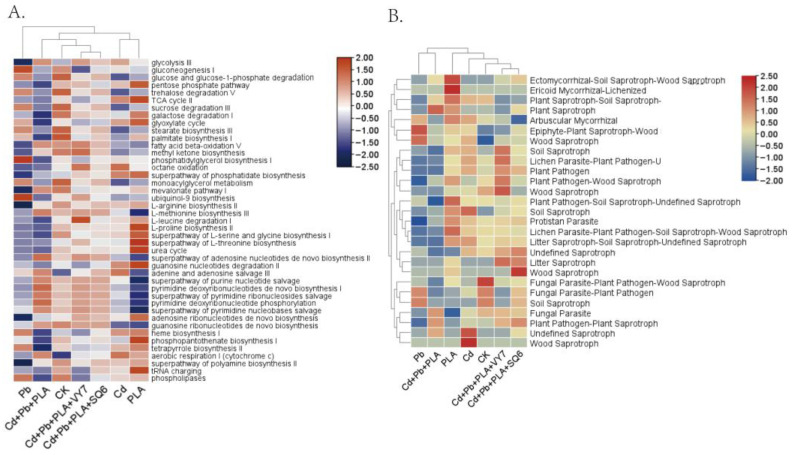
Predicted fungal functions. (**A**) Heatmap of fungal functions in different treatment groups. The *x*-axis represents treatment groups, and the *y*-axis represents functional genes. The color blocks on the left indicate the associated functions. (**B**) Functional classification of fungi using FUNGuild. The *x*-axis represents the treatment group, and the *y*-axis represents the nutritional regimen. The colour blocks on the left indicate relative abundance.

For fungal function, MetaCyc was used for metabolic pathway analysis, categorizing pathways into seven major classes: Carbohydrate Metabolism, Lipid Metabolism, Amino Acid Metabolism, Nucleotide Metabolism, Metabolism of Cofactors and Vitamins, Energy Metabolism and Respiration, and Cellular Processes (Figure 6A). The results suggested that the Cd treatment groups were associated with a decrease in the predicted abundance of pathways related to lipid metabolism and part of nucleotide metabolism, while showing an increase in the predicted abundance of pathways involved in energy metabolism and respiration. The Cd+Pb+PLA co-contamination group showed a general decrease in the predicted abundance of pathways related to carbohydrate, lipid, and amino acid metabolism. Compared with the Cd+Pb+PLA group, PGPB addition was associated with an increased predicted abundance of most metabolic pathways, except for nucleotide metabolism.

FUNGuild was used to annotate fungal trophic modes, classifying fungi into three categories: pathotroph, symbiotroph, and saprotroph. As shown in Figure 6B, the seven treatment groups clustered into two main branches: the Pb and Cd+Pb+PLA groups clustered together, while the remaining groups formed another cluster. In the Cd treatment groups, the relative abundance of saprotrophic fungi increased, whereas the PLA treatment was associated with an increased relative abundance of animal pathogen-related fungi.

### 3.9. Prediction of Nitrogen and Phosphorus Cycling Functions

**Figure 7:**
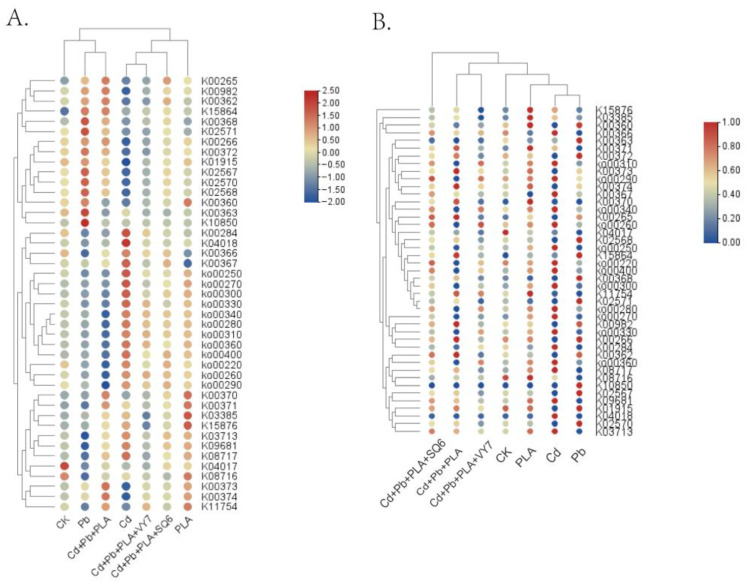
Heatmap of N (**A**) and P (**B**) cycling in different treatment groups. The *x*-axis represents the different treatment groups, and the *y*-axis represents the different genes.

As shown in Figure 7A, nitrogen metabolism-related KEGG Ortholog (KO) genes were categorized into four major pathways: nitrogen uptake and transport, nitrogen assimilation, nitrogen translocation and allocation, and nitrogen reutilization. Compared with the CK group, the Cd treatment group showed an increase in the predicted relative abundance of genes associated with amino acid metabolism related to nitrogen assimilation, while the Cd+Pb+PLA group exhibited a higher predicted abundance of genes involved in nitrogen assimilation pathways. Cluster analysis of nitrogen cycle-related genes indicated that the seven treatment groups were divided into two main clusters, with the CK group clustering with the Cd+Pb+PLA+SQ6 group, and the remaining groups forming another cluster. These results suggest that multi-metal and microplastic co-contamination may influence the potential for nitrogen cycling in soil microbial communities.

PICRUSt2 was also used to predict the relative abundance of key genes involved in phosphorus cycling, including pathways related to signal transduction, phosphorus uptake and transport, and organic phosphorus activation and utilization (Figure 7B). Except for a few genes, the CK group generally showed lower predicted gene abundance. In the Cd treatment group, genes associated with organic phosphorus activation showed an increased predicted abundance. Compared with the Cd+Pb+PLA group, PGPB-inoculated treatments were associated with a higher predicted abundance of genes related to organic phosphorus activation pathways, suggesting a potential role in enhancing the transformation of organic phosphorus into bioavailable forms. Cluster analysis grouped the PLA, Cd+Pb+PLA, and Cd+Pb+PLA+SQ6 treatments together, while the remaining groups formed another cluster, indicating that combined metal and microplastic contamination may alter the potential for phosphorus cycling in soil.

### 3.10. Correlation Analysis

**Figure 8:**
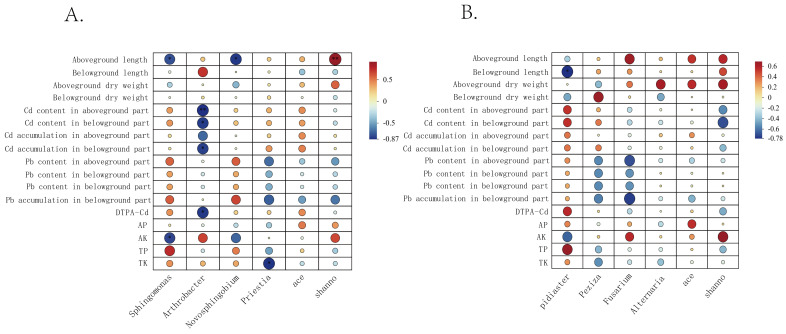
Relationships among spinach growth indices, soil chemical properties, bacterial communities (**A**); and fungal communities (**B**). The horizontal axis represents dominant microbial genera and diversity indices, while the vertical axis represents plant growth indices and soil chemical properties. Circle size and color intensity increase with the strength of the relationships. *, *p* < 0.05; **, *p* < 0.01.

Correlation analysis was performed to evaluate the relationships among important bacterial and fungal composition/diversity indices, spinach growth parameters, Cd/Pb accumulation, and soil chemical properties (Figure 8). Results showed that *Sphingomonas* abundance was positively correlated with shoot length (*p* < 0.05) and AK (*p* < 0.05). The bacterial community Shannon index was significantly positively correlated with shoot length (*p* < 0.01). *Olpidiaster* abundance was positively correlated with root length (*p* < 0.05), and *Novosphingobium* was positively correlated with shoot length (*p* < 0.05). *Fusarium* abundance was positively correlated with root Pb content (*p* < 0.05) and accumulation (*p* < 0.05). *Arthrobacter* abundance was positively correlated with plant Cd content (*p* < 0.01) and accumulation (*p* < 0.05), as well as soil DTPA-Cd (*p* < 0.05).

## 4. Discussion

### 4.1. Alleviation of Plant Growth Stress Under Dual Heavy Metal–MP Co-Contamination by PGPB

When plants are exposed to HM and MP pollution, the growth and development of shoots and roots are significantly inhibited [39]. MP-HM co-contamination may affect plant growth directly or indirectly. Previous studies have shown that single Cd or Pb pollution, and PLA MP pollution can cause stress on plant growth, potentially through mechanisms such as Cd/Pb-induced damage cell structures [40,41]; MP-HM complexes altering soil physicochemical properties, affecting soil porosity and structural stability, and reducing mineral elements; and changes the structure, composition, and function of rhizosphere microbial communities, impacting soil ecological stability [42,43]. In actual contaminated farmland, multiple heavy metals often coexist with MPs, forming complex pollution systems [44]. Therefore, this study investigated the effects of combined heavy metal (Cd, Pb) and MP (PLA) contamination on plant growth and found that Cd+Pb+PLA co-contamination significantly reduced shoot and root length as well as dry weight compared with individual Cd, Pb, or PLA treatments, indicating a pronounced synergistic effect. This may result from both the direct toxic effects of heavy metal ions and the indirect enhancement of heavy metal bioavailability and toxicity by MPs through altering soil properties and adsorbing/transporting heavy metals.

Under MP-HM co-contamination stress, inoculation with specific PGPB alleviated pollution-induced stress on plant growth and promoted nutrient uptake. PGPB play important roles in regulating plant growth and the soil ecological environment [45]. Studies have reported that PGPB can activate nutrients through their metabolic activities, including phosphate solubilization and nitrogen fixation, and secrete plant hormones to promote plant growth [46]. To our knowledge, this study is the first to explore the alleviating effect of PGPB on plant growth stress under dual heavy metal–MP co-contamination. Cd+Pb+PLA co-contamination reduced shoot and root length, whereas inoculation with PGPB capable of producing IAA and solubilizing phosphate may alleviate stress-induced damage through multiple pathways. PGPB inoculation increased soil available phosphorus and potassium contents, effectively mitigating the reduction in soil mineral elements caused by Cd+Pb+PLA co-contamination and improving the soil environment. Among plant hormones, IAA plays a significant role in plant growth, primarily by promoting root development [47]. *Bacillus* sp. SQ6 and *Enterobacter* sp. VY7 possess plant growth-promoting traits such as IAA production, and their inoculation increased shoot and root length. Previous research indicates that some metal-resistant PGPB can improve soil pollution conditions by releasing chelators and biosurfactants or by altering redox potential [48]. However, key parameters such as soil organic matter and microbial biomass carbon were not measured in this study, which may limit the interpretation of nutrient cycling processes. Future studies should incorporate these indicators to provide a more comprehensive understanding. Through biostabilization and synergistic regulation, PGPB reduce toxic effects on plants. Many PGPB can effectively adsorb heavy metal ions via functional groups on cell walls or secreted extracellular polymeric substances, forming a biological barrier [49], thereby reducing heavy metal ion accumulation in plants and alleviating stress. In this study, PGPB-inoculated groups showed a decreasing trend in Cd and Pb content in plant roots. PGPB may alleviate heavy metal and MP co-contamination stress on plant growth by adsorbing or precipitating Cd and Pb ions.

### 4.2. Effects of PGPB on Spinach Rhizosphere Microbial Community Composition and Structure

The structure, composition, and stability of rhizosphere microbial communities are crucial for ecosystem productivity, as they play irreplaceable roles in plant growth promotion and stress tolerance through participation in nutrient cycling (e.g., nitrogen fixation, phosphate solubilization, potassium mobilization, hormone production) [50]. Heavy metals such as Cd and Pb can affect their composition. The results of this study showed that pollution caused significant differences in dominant bacterial genera, such as *Sphingomonas*, *Novosphingobium*, *Flavisolibacter*, and *Rhizobium*, as well as fungal genera, including *Iodophanus* and *Lobulomycetales_gen_Incertae_sedis*, among different treatment groups, and reduced bacterial and fungal community diversity. This is consistent with the findings of Hou et al. Multiple studies have confirmed that Firmicutes and Pseudomonadota are tolerant to heavy metals and MPs, and they can reduce heavy metal-induced stress on plants through bioaccumulation and adsorption [51,52]. Therefore, regulating rhizosphere microbial community composition and structure may alleviate dual MP-HM co-contamination stress on plants. Using high-throughput sequencing, this study found that PGPB inoculation altered rhizosphere microbial community composition and structure, which is consistent with numerous previous studies indicating that PGPB are key drivers of rhizosphere microecological remodeling [53].

Existing studies have indicated that PGPB can regulate rhizosphere microbial composition and function, thereby mitigating damage caused by heavy metal and MP pollution [54,55,56]. In this study, strains SQ6 and VY7 significantly and differentially regulated rhizosphere bacterial and fungal community structures under Cd, Pb, and PLA co-contamination stress. Compared to the Cd+Pb+PLA co-contamination group, PGPB-inoculated treatments significantly increased the relative abundance of beneficial genera, such as *Sphingomonas* and *Novosphingobium*. Previous studies have confirmed that these bacteria can degrade complex organic matter and are associated with MP degradation. *Sphingomonas* has been reported as a heavy metal-tolerant bacterium widely present in heavy metal-contaminated soils [57,58]. Meanwhile, nitrogen-fixing genera such as *Rhizobium* and *Devosia*, as well as potentially growth-promoting genera such as *Nocardioides*, also showed increasing trends in relative abundance [59].

Previous studies on PGPB-mediated alleviation of MP–HM co-contamination have mainly focused on bacterial communities, while fungal components have often been overlooked. This study demonstrated that under Cd+Pb+PLA co-contamination, PGPB inoculation increased the abundance of certain beneficial fungi like *Mortierella* and *Arthrobotrys*. Research indicates these fungi are associated with organic matter decomposition and nematode-trapping, respectively, thereby enhancing fungal community function and composition [60,61]. Furthermore, fungal community structure and diversity in soils contaminated with HMs and MPs are altered, with certain tolerant or plastic-degrading fungi becoming dominant taxa [62]. *Fusarium* is a widespread filamentous fungus and an important model for biological control research [63]. It can cause plant wilt, invade roots causing rot, and its secondary metabolites like zearalenone and fumonisins harm plants and human health [64,65]. In this study, the relative abundance of *Fusarium* was higher in polluted treatments but was suppressed in PGPB-inoculated treatments. This suggests PGPB strains may effectively inhibit pathogen proliferation through antimicrobial production, competition, or inducing plant systemic resistance [66].

Diversity indices provide a useful representation of microbial community responses to environmental stress and are important for monitoring soil health and assessing ecological risks [67]. Previous studies have shown that soil bacterial community diversity generally shows a declining trend under heavy metal pollution [68]. Heavy metal ions can damage cell membranes, denature proteins, and interfere with enzyme activity, leading to the death of sensitive microorganisms [69]. The decline of sensitive taxa and the dominance of tolerant species alter community structure. In this study, PGPB inoculation increased bacterial community diversity indices, possibly by immobilizing heavy metals or secreting metabolites to alleviate pollution-induced stress to the rhizosphere environment. The increased diversity indices in PLA-added groups may be related to the complex mechanisms of MPs. MP surfaces can serve as a novel habitat distinct from bulk soil, known as the plastisphere, which may initially attract microbial colonization and increase community richness. Under Cd+Pb+PLA co-contamination, PGPB inoculation generally increased bacterial and fungal diversity indices. This is consistent with previous findings that PGPB alleviate pollution-induced inhibition of bacterial communities and suppress the proliferation of pathogenic fungi through competition and antagonism, thereby restoring rhizosphere microbial communities toward a stable ecological state [70]. Fungal communities may possess greater buffering capacity, resulting in smaller changes in diversity indices under pollution compared to bacteria. Correlation analysis revealed positive correlations between bacterial and fungal community ACE and Shannon indices and plant growth, as well as soil AP and AK, indicating that PGPB alleviate Cd+Pb+PLA co-contamination stress by regulating microbial community composition.

### 4.3. Functional Prediction and Nitrogen-Phosphorus Cycling

One important pathway by PGPB may alleviate MP–HM co-contamination stress is through improving soil mineral nutrient availability, which is often reduced under such conditions. In this study, Cd+Pb+PLA co-contamination significantly decreased AK compared to single pollution treatments, whereas strains VY7 and SQ6 increased soil mineral nutrient levels. However, microbial functional responses to combined MP–HM contamination, particularly those related to nutrient cycling, remain insufficiently explored.

Linking microbial community composition with functional prediction based on KEGG annotation provides insights into potential ecological functions and their responses to environmental changes [71]. Genes associated with nitrogen and phosphorus cycling represent key components of these predicted functional profiles. Previous studies have suggested that Cd contamination is associated with an increased predicted abundance of genes across several functional categories, including cellular processes, environmental information processing, and metabolism, indicating potential adaptive responses of microbial communities to heavy metal stress. In particular, genes related to transport proteins may show higher predicted abundance, which could be associated with microbial strategies for reducing intracellular metal toxicity [72]. Similarly, metabolic pathways related to stress adaptation may exhibit increased predicted functional potential, potentially supporting cellular maintenance and detoxification processes [73,74].

Bacteria are generally considered more sensitive to environmental stress than fungi [75], and may respond rapidly through shifts in community composition and functional potential [76]. In contrast, fungi may contribute to community stability by adjusting metabolic strategies. In this study, saprotrophic fungi showed increased relative abundance under heavy metal stress, which may be associated with their role in organic matter decomposition and nutrient supply. Previous studies suggest that some saprotrophic fungi possess strong metal tolerance and may adjust membrane-related metabolic processes to cope with stress [77]. Overall, bacteria and fungi may contribute to ecosystem stability under MP–HM co-contamination through distinct but complementary ecological strategies.

The nitrogen cycle includes key microbial processes such as nitrogen fixation, ammonification, nitrification, and denitrification [78]. Nitrifying microorganisms are known to be sensitive to heavy metals [79], and co-contamination may therefore reduce their relative abundance and functional potential, potentially limiting nitrification processes. In addition, genes encoding key enzymes involved in nitrogen transformation (e.g., urease and nitrate reductase) may show altered predicted abundance, which could influence nitrogen availability for plant uptake [80]. PGPB inoculation has been reported to influence the predicted abundance of genes related to nitrogen transformation, potentially contributing to improved nitrogen utilization [81].

Similarly, most soil phosphorus exists in organic forms and requires microbial mineralization to become bioavailable [82]. Functional prediction in this study suggested that genes associated with organic phosphorus activation exhibited increased predicted abundance under certain treatments. Compared with the Cd+Pb+PLA group, PGPB inoculation was associated with higher predicted abundance of genes involved in phosphorus activation pathways, suggesting a potential role in enhancing phosphorus availability.

Overall, the results indicate that PGPB inoculation may influence the predicted functional potential of microbial communities, particularly in pathways related to nutrient cycling and stress adaptation. These changes may contribute to improved soil ecological function and plant growth under MP–HM co-contamination. However, it should be noted that functional predictions based on 16S rRNA gene data have inherent limitations in accuracy and resolution. Future studies should incorporate more precise approaches, such as metagenomic sequencing and quantitative PCR (qPCR), to validate functional profiles and obtain more robust and reliable insights into microbial functions regulated by PGPB in MP-HM co-contaminated soils.

## 5. Conclusions

This study demonstrates that combined contamination by Cd, Pb, and PLA MPs significantly inhibits spinach growth, disrupts soil nutrient availability, and alters rhizosphere microbial community structure. Inoculation with PGPB effectively mitigated these adverse effects by enhancing plant biomass, improving soil available nutrients (AP and AK), and reducing heavy metal accumulation in plant tissues. Moreover, PGPB reshaped both bacterial and fungal communities, increased microbial diversity, and promoted the enrichment of beneficial taxa associated with stress resistance and nutrient transformation. Functional predictions further revealed that PGPB enhanced key metabolic processes, particularly those involved in nitrogen and phosphorus cycling, thereby contributing to improved soil ecological function and system stability under co-contamination stress. Overall, these findings highlight the important role of PGPB in regulating plant–soil–microbe interactions under complex pollution scenarios and demonstrate their potential as a sustainable strategy for remediation. However, the concentrations of MPs and HMs used in this study may exceed typical field levels. Additionally, as plant species differ in root architecture and growth cycle, rhizosphere responses may vary in cereals or root crops compared with leafy vegetables like spinach. Future research should incorporate environmentally relevant and gradient contaminant concentrations, as well as field-based experiments, long-term assessments, and mechanistic studies across diverse plant types to evaluate the stability, effectiveness, and ecological safety of PGPB applications in multi-metal–microplastic composite pollution agroecosystems.

## Figures and Tables

**Table 1:** Design of experiments.

Experimental Treatment	MPs	MP Concentrations/% (*w*/*w*)	Cd Concentrations/mg·kg^−1^	Pb Concentrations/mg·kg^−1^
CK	0	0	0	0
Cd	0	0	10	0
Pb	0	0	0	200
PLA	PLA	0.5%	0	0
Cd+Pb+PLA	PLA	0.5%	10	200
Cd+Pb+PLA+SQ6	PLA	0.5%	10	200
Cd+Pb+PLA+VY7	PLA	0.5%	10	200

**Table 2:** Physicochemical properties and available metal contents of soil under different treatments (mean ± SD).

Treatment	AP (mg·kg^−1^)	AK (mg·kg^−1^)	TP (mg·kg^−1^)	TK (mg·kg^−1^)	DTPA-Cd (mg·kg^−1^)	DTPA-Pb (mg·kg^−1^)
CK	37.07 ± 7.01 bc	151.8 ± 19.08 a	1.50 ± 0.00 b	7.55 ± 0.04 b	-	-
Cd	24.28 ± 0.19 d	133.35 ± 1.60 c	0.95 ± 0.01 c	5.94 ± 0.01 d	2.09 ± 0.01 b	-
Pb	30.41 ± 23.56 cd	143.85 ± 0.88 b	1.26 ± 0.00 b	7.71 ± 0.03 ab	-	15.35 ± 0.04 b
PLA	40.14 ± 0.02 b	153.30 ± 0.24 a	1.47 ± 0.02 b	7.44 ± 0.00 b	-	-
Cd+Pb+PLA	37.25 ± 2.62 bc	114.35 ± 0.20 e	1.90 ± 0.02 a	7.93 ± 0.00 a	2.49 ± 0.00 ab	17.25 ± 0.33 a
Cd+Pb+PLA+VY7	47.70 ± 18.20 a	137.15 ± 7.48 c	1.49 ± 0.03 b	7.49 ± 0.00 b	2.83 ± 0.00 a	16.77 ± 0.15 a
Cd+Pb+PLA+SQ6	35.27 ± 4.24 bc	126.40 ± 1.50 d	1.45 ± 0.01 b	6.92 ± 0.08 c	2.82 ± 0.12 a	17.10 ± 0.11 a

Different letters indicate significant differences between the treatments (*p* < 0.05).

## Data Availability

The data that support the findings of this study are available on request from the corresponding author.
